# A lack of race and ethnicity data in the treatment of hereditary hemorrhagic telangiectasia: a systematic review of intravenous bevacizumab efficacy

**DOI:** 10.1186/s13023-022-02371-0

**Published:** 2022-06-13

**Authors:** Panagis Galiatsatos, Cheri Wilson, Jaime O’Brien, Anna J. Gong, Dylan Angiolillo, James Johnson, Carlie Myers, Sara Strout, Stephen Mathai, Gina Robinson, Nicholas R. Rowan, Clifford R. Weiss

**Affiliations:** 1 Division of Pulmonary and Critical Care Medicine, Johns Hopkins School of Medicine, Baltimore, MD USA; 2 The Center of Clinical Excellence for Hereditary Hemorrhagic Telangiectasias at the Johns Hopkins Hospital, Baltimore, MD USA; 3 Office of Diversity, Inclusion, and Health Equity, Baltimore, MD USA; 4 Medicine for the Greater Good, Johns Hopkins School of Medicine, 4940 Eastern Avenue, 4th Floor, Asthma and Allergy Building, Baltimore, MD 21224 USA; 5 Division of Anesthesiology and Critical Care Medicine, Johns Hopkins School of Medicine, Baltimore, MD USA; 6 Albert Gnaegi Center for Health Care Ethics, Saint Louis University, Saint Louis, MO USA; 7 Department of Pharmacy, Johns Hopkins Hospital, Baltimore, MD USA

**Keywords:** Hereditary hemorrhagic telangiectasia, Bevacizumab, Race, Health equity

## Abstract

**Background:**

For extreme hereditary hemorrhagic telangiectasia (HHT) disease, treatments such as intravenous bevacizumab are often utilized. However, whether its efficacy is similar across diverse races and ethnicities is unclear.

**Methods:**

In this systematic review, we performed a search for English-language articles identified through PubMed, Embase, and Scopus databases whose research occurred in the United States (US). Search terms related to HHT, epistaxis, and intravenous bevacizumab. We searched specifically for the intervention of intravenous bevacizumab because the term serves as a suitable surrogate to convey a patient who has both a diagnosis of HHT and established care. We focused on number of patients recruited in intravenous bevacizumab trials who were identified by race or ethnicity.

**Results:**

Our search identified 79 studies, of which four were conducted in the US. These four were selected for our systematic review. In these studies, 58 total patients were evaluated (ranging from 5 to 34 participants), whereby, information on age and gender were included. However, none of the US-based studies shared race or ethnicity data.

**Conclusion:**

Inability to find studies regarding intravenous bevacizumab use in patients with HHT in which race and ethnicity are reported limits our ability to understand the therapy’s efficacy in specific populations. Without emphasis on race and ethnicity in such trials, showing the potential of HHT-related diversity in individuals with this disease may reaffirm implicit bias around HHT diagnosis and treatment. Future work on HHT should emphasize sociodemographic data collection and reporting in an effort to understand this disease in diverse populations.

**Supplementary Information:**

The online version contains supplementary material available at 10.1186/s13023-022-02371-0.

## Introduction

As an autosomal dominant genetic disease, hereditary hemorrhagic telangiectasia (HHT) has an estimated prevalence of 1 in 5000 individuals [1, 2]. HHT is characterized by clinically significant malformations of the vascular system that affect the mucous membranes of the nose and gastrointestinal system; however, it also impacts the central nervous, pulmonary, and hepatic systems [3]. The diagnosis of HHT relies heavily on clinical symptoms of epistaxis and presence of vascular malformations, specifically arteriovenous malformations (AVMs) and telangiectasias [2]. Despite the presence of these symptoms, HHT is still difficult to diagnosis, often delayed are missed altogether [4]. If the diagnosis is confirmed, a variety of clinical care, screenings, and potential local or systemic interventions are allocated to the patient in an effort to curb the dire impact of AVMs. However, while these HHT-related clinical services are vital, it is unclear if there are differences in receipt and/or outcomes in HHT patients across diverse sociodemographic factors, similar to many common pathologies, specifically in the United States [5–7]. Or does the rarity of the disease behave in an ability to control contextual factors from influencing patient outcomes resulting in significant differences and potential disparities? Therefore, evaluating HHT-related therapy in diverse patient populations in the United States should be made a priority to determine if such differences and disparities exist.

The aim of this study was to perform a systematic review of patients diagnosed with HHT who received intravenous bevacizumab to reduce HHT-related bleeding and describe their outcomes based on race and ethnicity. We chose to evaluate intravenous bevacizumab, as this therapeutic is reserved for severe HHT-related disease [2]. Hence, we considered it a reasonable surrogate to ensure that study patients were both diagnosed with HHT and managed by HHT-themed centers. Studies identified in our search would then enable us to identify whether gaps are present in patient representation based on sociodemographic variables.

## Methods

### Data sources

We performed a search for English-language articles in PubMed, Embase, and Scopus databases that encompassed the years 2000 to 27 July 2021 using the PICO framework [8]. We examined specifically the intervention of intravenous bevacizumab as a surrogate to convey that patients had both a diagnosis of HHT and established care. Search terms are available to review in the Additional file 1.

### Eligibility criteria

Studies were included if they explicitly examined the effects of intravenous bevacizumab use on HHT-related bleeding and reported data characterizing each participant’s race and/or ethnicity. We aimed for studies that occurred in the United States, where race and ethnicity are consistently found to be associated with variation in health outcomes [9]. Case reports (reporting of only one patient) were excluded. We excluded studies that did not specify how bevacizumab was administered (e.g., intranasal or intravenous), and we also excluded animal studies.

### Study identification

Three team members (PG, DA, JJ) finalized the research criteria, and two (DA, JJ) reviewed all titles and abstracts identified from the search strategy. Two individuals (DA, JJ) reviewed the full texts, and a third (PG) confirmed the inclusion or exclusion of a study.

### Data extraction

Data collected from each paper included number patients, severity of HHT-related disease, time span of the study, and, when available, timing of HHT diagnosis and HHT-related complications in the patients. Additional variables extracted included age, gender, and race and/or ethnicity.

### Outcome measures

We focused on number of patients recruited into intravenous bevacizumab trials who were identified by race or ethnicity. Secondary outcomes explored patient outcomes, categorized by race and ethnicity.

### Data synthesis and analysis

We examined the following comparisons in regard to intravenous bevacizumab treatment for HHT-related epistaxis: number of patients recruited by race and ethnicity with outcomes characterized by differences in race or ethnicity.

### Qualitative analysis

We used a narrative summary approach in our description of study characteristics and variation in quality indicators among identified studies. Further, we evaluated how these factors might affect our understanding of HHT diagnosis in the identified studies included in this review.

## Results

### Study selection

**Fig. 1:**
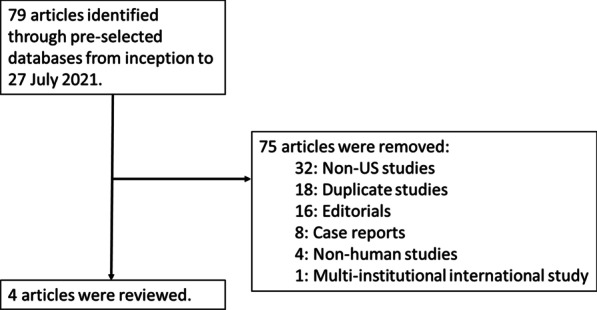
Flow chart showing selection process of articles for the systematic review

The search identified 79 studies of possible relevance. Of these studies, four adhered to the predefined selection criteria and were included for review. Studies were evaluated for publication bias. Figure 1 shows the results of the search strategy as well as reason for study selection removal.

### Study description

Four studies were from North America (United States) and totaled 58 patients (ranging from 5 recruited patients to 34) who were administered intravenous bevacizumab for HHT that had contributed to refractory anemia as a result of epistaxis or gastrointestinal bleeding [10–13]. Thompson et al [10] published a prospective, open-label, non-comparative study at a single institution from 2012 to 2013 for the treatment of severe epistaxis. Iyer et al [11] conducted a retrospective study in which they reviewed charts of patients who received intravenous bevacizumab from 2013 to 2017 at a single institution. In that study, 15 patients had severe epistaxis, 4 had severe gastrointestinal bleeding, and 15 had both [11]. Epperla et al [12] reviewed the charts of patients who received treatment from 2009 to 2014 at a single institution for epistaxis (4 patients) and gastrointestinal bleeding (1 patient). Al-Samkari et al [13] conducted a retrospective analysis of patients with severe epistaxis (3 patients), gastrointestinal bleeding (2 patients), or both (8 patients) at a single institution from 2015 to 2018.

Outcomes measured included severity of epistaxis [10, 11, 13], quality of life [10, 11], monitoring of hemoglobin [10, 12, 13], monitoring of packed red blood cell transfusions [10, 12], monitoring of iron infusions [13], and number of hospitalizations. [12]

### Sociodemographic variables

All four HHT studies reported age and gender in regard to the efficacy of intravenous bevacizumab. The median age range for the patients in the four studies was 61 years (range, 46–67 years) [10], 63 (range, 57–72 years) [11], 54 (range, 43–61 years) [12], and 60 (range, 49–81 years) [13]. Of all 58 patients recruited for these four studies, 30 (52%) were female: 3 (50%) [10], 21 (62%) [11], and 6 (46%) [13]. One study had only male patients. [12]

Two studies conducted genetic mutation analysis related to HHT [11, 13]. Iyer et al [11] conducted genetic mutation analysis in 20 of their 34 participants, and Al-Samkari et al [13] conducted the analysis in 5 of their 13 participants.

None of the four studies reported race or ethnicity in their findings. Epperla et al [12] mentioned that race information was collected, but it was not reported in the patients’ sociodemographic.

## Discussion

In this systematic review of intravenous bevacizumab for patients with established HHT, we identified a significant gap in presenting sociodemographic variables that identify race and ethnicity of each participant. Though we noted an emphasis on participant gender and age, as well as genetics and genotypes, in the studies reviewed, the lack of racial and ethnicity information should sound an alarm around the context of health equity’s role in the management of patients with HHT. Further, the influence of genes in health and health outcomes is known to be affected by nonbiological factors that create various and unique phenotypes in patients seeking medical care. Without factors of race and ethnicity captured in these studies, the benefits to HHT phenotypes from such interventions will remain unclear, resulting in a significant barrier to creating more precise medical therapies.

With rare genetic diseases, sociodemographic variables may not receive as much focus as the genes that cause the disease itself. However, if such attention is not emphasized, it will be impossible to understand how different populations are impacted by the genetic disorders. Disparities in health status, especially in the United States, are largely the result of longstanding, pervasive discrimination that is based on race and ethnicity [14]. In the case of common noncommunicable chronic diseases, minority populations identified by race and ethnicity continue to have poorer health outcomes than other populations, even given similar sociodemographic variables, despite substantial advancements in medical therapies for these diseases (e.g., heart failure, diabetes) [15]. These inequities have historically been an issue in clinical research owing to a lack of diversity in subject population recruitment for treatment studies [16] and non-reporting of sociodemographic factors [17]. Both of these deficiencies have the potential to delay, disrupt, or disable minority populations from obtaining the proper medical diagnosis and effective treatment.

Clinical trials in the United States have been cited previously for their lack of racial and ethnic diversity [18, 19]. As such, a significant proportion of Americans may be exposed to intervention untested in their populations. In response to this dearth of diversity, the National Institutes of Health in the United States mandated the inclusion of minority populations, a mandate aimed to promote fairness in the research process and inclusiveness [20, 21]. While this mandate likely played a crucial role as a catalyst, health differences and disparities experienced by minority patients persist [20]. To assure interventions are effective for all patients across diverse populations, research is needed that is group-specific or subgroup-reviewed [22]. Therefore, at a time where patients are encouraged in making treatment decisions along with their clinicians, it may not be surprising that hesitation is often seen in patients where there is a deficit in appropriate information needed to answer patient questions regarding treatment outcomes on similar persons [23]. As advancements are made in the treatment of patients with HHT-related complications, it will be unclear if all HHT patients will benefit equally. We emphasized that HHT diagnosis may be both underrepresented and delayed. However, we cannot be certain how such factors affect HHT-related outcomes across a diverse patient population in the absence of race and ethnicity data. Further, the lack of information related to race and ethnicity in these studies of intravenous bevacizumab, a treatment reserved for severe HHT cases, spotlights such a moral and ethical concern.

A potential concern for HHT and its impact on diverse populations may also reside in its ability to diagnose the disease itself. To assist clinicians in the diagnosis of HHT, the Curacao Criteria were established in 2000. These criteria focus on epistaxis, family history of HHT, presence of AVMs on organs, and telangiectasias [24]. Telangiectasia lesions usually appear during adulthood and are commonly seen on mucous membranes, as well as on the face and distal extremities [3]. These telangiectasias represent AVMs of the dermal microvasculature, which explains their tendency to bleed. The telangiectasias are dark red in color and at times are raised. The emphasis of the Curacao Criteria to rely on findings of telangiectasia may raise concern in individuals with darker skin, in whom identifying telangiectasias may be more difficult. Depending on visual appearance would be in accordance with racial disparities seen in dermatological disorders and medical measurements that rely on skin pigmentation [25, 26], and could delay HHT diagnosis in certain patient populations. Delayed diagnosis may cause a delay in treatment, confounding the potential impact of HHT-related therapies in specific demographics. Therefore, investigating whether certain populations are disproportionately impacted by HHT-related treatment is a logical extension to investigate HHT-related difference, and potential disparities.

Emphasizing race and ethnicity may also provide an additional benefit regarding concerns around potential bias with the diagnosis of HHT. The tendency for stereotype-confirming thoughts to occur outside conscious awareness is known as implicit bias [27]. Just as implicit bias has found its way into medical practice, education, and publications before [28], it may have also established an unwanted presence in the diagnosis of HHT. Among the studies that our search identified, classification by country of origin showed that many were from nations composed predominantly of pale skin, White persons (United States, France, Denmark) and had established HHT-related databases for monitoring their patients. In contrast, in countries with dark-skinned persons, HHT diagnoses continue to be discussed as novel case reports [29, 30]. The implicit bias may come from the need for a clinical diagnosis of HHT to rely on family history and mucosal and/or dermatological findings, as previously mentioned [2]. If HHT is a disease that is underreported in its diagnosis [2], the underreporting is likely worse in some populations than in others. This disparity may manifest as a delay in diagnosis and a delay or even failure to treat with a drug such as intravenous bevacizumab. This undertreatment would, in turn, limit our ability to understand the therapy’s efficacy in diverse populations.

The implicit bias in HHT diagnosis may be present for several reasons. For instance, in regard to dermatological findings and diseases such as in melanoma, minority populations often present later than non-minority populations and have late-stage complications [31–33]. Although many reasons may account for this health disparity (e.g., access to care), physician awareness of the disease and difficulty in physical exam findings due to the lack of inclusive data available may contribute substantially [34]. A lack of disease awareness may also contribute to unknown family histories of HHT. Without knowledge of family history, clinical diagnosis of this genetic disease can be much more difficult. However, even with the potential for such implicit bias to create disparities in the diagnosis of HHT, it is unclear if underdiagnosis in certain populations results in variable efficacy of HHT-related therapies based on race and ethnicity because these variables are not reported in such clinical trials.

There are a several limitations to address. First, limiting to only the United States for intravenous bevacizumab outcomes in HHT resulted in excluding many studies coming from HHT centers throughout the world. However, this was a decision in an effort to review the sociodemographic variable of race, a complex and multidimensional construct, as defined by standards set forth in the United States health services [35]. Second, we focused on intravenous bevacizumab only, which in of itself is often seen as a therapeutic of “last resort” for HHT-related complications. Therefore, the notion of race in HHT studies in the United States may be well recorded in other HHT-related interventions and trials, warranting exploration. Finally, there are contextual-level sociodemographic variables that impact health outcomes (e.g. neighborhood poverty levels) that were not explored in this review and are known to have an effect on the weight of race and its influence on health outcomes. Further explorations are warranted in such future reviews for HHT studies.

## Conclusion

The inability to find studies on the efficacy of intravenous bevacizumab that include data on patient race and ethnicity is concerning for the ability to identify and ameliorate potential health equity gaps. Further, without emphasis on race and ethnicity in such trials, showing the potential of HHT-related diversity in individuals with this disease may reaffirm implicit bias around HHT diagnosis and resulting treatment. Therefore, an emphasis must be placed on ensuring that HHT treatment centers and databases capture all sociodemographic variables from the patients they care for, and share such data when reporting research outcomes and findings. We stress the importance of the structural and institutional inequities that have become inherently intertwined with health outcomes in genetic diseases in an attempt to combat the pervasive history of pathologizing minority groups via a fixed focus on genetic expression alone. Only then can we identify HHT-related disparities and work collaboratively to mitigate them.

## Supplementary Information

**Additional file 1.** Terms used for performing systematic review.

## Data Availability

Being a systematic review, all studies included are cited and can be found publicly. Our algorithm for the systematic review can be found in the manuscript; additional information can be provided by request to the corresponding author.

## Abbreviations

AVM: Arteriovenous malformation
HHT: Hereditary hemorrhagic telangiectasia
