# Supplementary material for: A lack of race and ethnicity data in the treatment of hereditary hemorrhagic telangiectasia: a systematic review of intravenous bevacizumab efficacy
Source: Orphanet J Rare Dis. 2022 Jun 13;17:220. doi: 10.1186/s13023-022-02371-0 (PMC9195340; doi:10.1186/s13023-022-02371-0)
Supplement: Supplementary file 1 — Additional file 1. Terms used for performing systematic review. [file 13023_2022_2371_MOESM1_ESM.docx]

**Supplement**. Search results based on databases.

| **Database** | **Search Terms** | **Number of Studies Identified** |
| --- | --- | --- |
| **PubMed** | | |
| ***With Race, Ethnicity Terms*** | (((race[MeSH Terms]) OR (racial factors[MeSH Terms]) OR (continental population groups[MeSH Terms]) OR ("race"[Text Word]) OR ("racial factors"[Text Word]) OR ("continental population groups"[Text Word]) OR ("ancestry groups"[Text Word])) AND ((((telangiectasia, hereditary hemorrhagic[MeSH Terms]) OR (hereditary hemorrhagic telangiectasia[MeSH Terms]) OR (telangiectasia, hereditary hemorrhagic, of rendu, osler, and weber[MeSH Terms]) OR ("telangiectasia, hereditary hemorrhagic"[Text Word]) OR ("hht"[Text Word]) OR ("hereditary hemorrhagic telangiectasia"[Text Word]))) AND (((epistaxis[MeSH Terms]) OR (nosebleed[MeSH Terms]) OR ("epistaxis"[Text Word]) OR ("nosebleed"[Text Word])) AND (("bevacizumab"[Text Word]) OR (bevacizumab[MeSH Terms])))) | 0 |
| ***Without Race, Ethnicity Terms*** | (((telangiectasia, hereditary hemorrhagic[MeSH Terms]) OR (hereditary hemorrhagic telangiectasia[MeSH Terms]) OR (telangiectasia, hereditary hemorrhagic, of rendu, osler, and weber[MeSH Terms]) OR ("telangiectasia, hereditary hemorrhagic"[Text Word]) OR ("hht"[Text Word]) OR ("hereditary hemorrhagic telangiectasia"[Text Word]))) AND (((epistaxis[MeSH Terms]) OR (nosebleed[MeSH Terms]) OR ("epistaxis"[Text Word]) OR ("nosebleed"[Text Word])) AND (("bevacizumab"[Text Word]) OR (bevacizumab[MeSH Terms]))) | 91 |
| **Embase** | | |
| ***With Race, Ethnicity Terms*** | ('ancestry group'/exp OR 'race'/exp) AND 'rendu osler weber disease'/exp AND 'epistaxis'/exp AND 'bevacizumab'/exp | 2 |
| ***Without Race, Ethnicity Terms*** | 'rendu osler weber disease'/exp AND 'epistaxis'/exp AND 'bevacizumab'/exp | 186 |
| **Scopus** | | |
| ***With Race, Ethnicity Terms*** | ( ALL ( "epistaxis"  OR  "nosebleeds" )  AND  ALL ( "hereditary hemorrhagic telangiectasia"  OR  "HHT"  OR  "rendu osler weber disease" )  AND  ALL ( "race"  OR  "race factors"  OR  "continental population groups"  OR  "ancestry groups"  OR  "race ifference" )  AND  ALL ( "bevacizumab" ) ) | 3 |
| ***Without Race, Ethnicity Terms*** | ( ALL ( "epistaxis"  OR  "nosebleeds" )  AND  ALL ( "hereditary hemorrhagic telangiectasia"  OR  "HHT"  OR  "rendu osler weber disease" )  AND  ALL ( "bevacizumab" ) ) | 459 |
